# Maternal obesity, gestational diabetes mellitus, and diet in association with neurodevelopment of 2-year-old children

**DOI:** 10.1038/s41390-022-02455-4

**Published:** 2023-01-03

**Authors:** Lotta Saros, Annika Lind, Sirkku Setänen, Kristiina Tertti, Ella Koivuniemi, Annarilla Ahtola, Leena Haataja, Nitin Shivappa, James R. Hébert, Tero Vahlberg, Kirsi Laitinen

**Affiliations:** 1 Institute of Biomedicine, Research Centre for Integrative Physiology and Pharmacology, University of Turku, 20520 Turku, Finland; 2 Turku Institute for Advanced Studies (TIAS), University of Turku, 20500 Turku, Finland; 3 Department of Psychology, University of Turku, 20500 Turku, Finland; 4 Department of Psychology, Åbo Akademi University, 20500 Turku, Finland; 5 Department of Pediatric Neurology, University of Turku and Turku University Hospital, 20520 Turku, Finland; 6 Department of Obstetrics and Gynecology, University of Turku and Turku University Hospital, 20520 Turku, Finland; 7 Department of Psychology and Speech-Language Pathology, University of Turku, 20500 Turku, Finland; 8 Department of Pediatric Neurology, Children’s Hospital and Pediatric Research Center, University of Helsinki and Helsinki University Hospital, 00290 Helsinki, Finland; 9 Cancer Prevention and Control Program and Department of Epidemiology and Biostatistics, Arnold School of Public Health, University of South Carolina, Columbia, SC USA; 10 Department of Nutrition, Connecting Health Innovations LLC, Columbia, SC USA; 11 Institute of Clinical Medicine, Biostatistics, University of Turku, 20014 Turku, Finland; 12 Functional Foods Forum, University of Turku, 20014 Turku, Finland

## Abstract

**Background:**

Maternal metabolic disturbances and diet may influence long-term infantile neurodevelopment. We investigated whether maternal gestational diabetes mellitus (GDM), obesity, and diet could affect the neurodevelopment of 2-year-old children.

**Methods:**

Neurodevelopment of children (*n* = 243) born to mothers with overweight or obesity was assessed with the Bayley Scales of Infant and Toddler Development–Third Edition, and the Hammersmith Infant Neurological Examination. Maternal adiposity was determined by air displacement plethysmography, and GDM with an oral glucose tolerance test. Dietary assessment included diet quality and fish consumption questionnaires, and three-day food diaries, from which dietary inflammatory index (DII^®^) scores were computed.

**Results:**

GDM was associated with weaker expressive language skills (adj.*β* = −1.12, 95% CI = −2.10;−0.15), and higher maternal adiposity with weaker cognitive, language, and motor skills in children (adj.*p* < 0.05). Maternal good dietary quality (adj.*β* = 0.87, 95% CI = 0.004;1.73) and higher fish consumption (adj.*p* = 0.02) were related to better expressive language skills. DII scores were not associated with children’s neurodevelopment.

**Conclusions:**

Findings suggest that GDM and higher maternal adiposity may lead to weaker neurodevelopmental skills, although still within the mean normative range in this population of children. Good dietary quality and higher fish consumption during pregnancy could benefit a child’s language development.

**Impact:**

- Gestational diabetes mellitus and maternal higher adiposity may have unfavorable effects on a 2-year-old child’s neurodevelopment.
- An overall good quality of diet and higher fish consumption during pregnancy may result in more favorable cognitive and language skills when the child is 2-year-old.
- Our findings reveal that women with overweight or obesity, a risk group for pregnancy complications, could benefit from dietary counseling to support their children’s neurodevelopment.

## Introduction

The global burden of obesity has increased over the past few decades,^1^ and is especially alarming in women of childbearing age.^2^ Over two fifth Finnish pregnant woman (41.9%) had overweight or obesity (body mass index, BMI 25–29.9 kg/m^2^ and ≥30 kg/m^2^, respectively) in 2019.^3^ Pregnant women with overweight or obesity are more prone to develop metabolic aberrations including gestational diabetes mellitus (GDM) and birth complications compared to women with normal weight (BMI 18–24.9 kg/m^2^).^4,5^ It is possible that the environment during pregnancy, including obesity and GDM, can affect the long-term neurodevelopment of children through foetal programming,^6^ by triggering changes in fetal brain development, thus disturbing optimal growth and development in the uterus.^7^

Previous evidence has linked GDM with poorer mental and memory performance in 1-year-old children,^8^ mild cognitive impairment in school-age children,^9^ and poorer language functions in 1.5- and 7-year-old children.^10^ However, not all investigators have detected an association between GDM or hyperglycemia and the child’s neurodevelopmental variables.^11–13^ The impact of maternal obesity and weight gain during pregnancy on the child’s neurodevelopment has been evaluated in one meta-analysis, including 32 studies,^14^ where higher maternal BMI increased the risk of developmental delay, emotional, and behavioral problems in children.^14^ Nonetheless, a recent study identified no association between pre-pregnancy BMI and neurodevelopment in 4-year-old children.^13^ As the findings are not completely consistent, further research is necessary to establish the extent to which maternal metabolic conditions can affect the child’s neurodevelopment.

Maternal diet, a modifiable lifestyle factor, is the primary source of nutrients for the foetus.^15^ Thus, it is another contributor to child neurodevelopment. Previous evidence from a meta-analysis, including 16 studies, indicated that a good-quality diet, i.e., high consumption of foods like fish, whole-grains, vegetables, and fruits during pregnancy may have small beneficial effects on the child’s cognitive functions.^16^ In addition to dietary quality, dietary patterns have been linked with neurodevelopment. In one study three dietary patterns were identified during pregnancy, the “meat + potatoes” and “white bread + coffee” patterns associated with lower intelligence quotients in 1-year-old children compared to the “fruit + vegetables” pattern.^17^ Furthermore, the role of individual nutrients in the maternal diet are of interest with regard to neurodevelopment. Particularly n-3 polyunsaturated fatty acids (PUFA), eicosapentaeonic acid (EPA) and docosahexaenoic acid (DHA), derived from fish are pivotal for the child’s brain development.^18^ Indeed, fish consumption and an overall n-3 PUFA intake during pregnancy associate with better visual development, problem solving and motor skills in 6-, 12- and 24-month-old children.^19,20^ Along with cognition, behavioral problems have been investigated with regard to the maternal diet. One study reported that the maternal intake of alpha-linolenic acid, n-6 PUFA, and monounsaturated fatty acids (MUFA), increased the risk for emotional problems in 5-year-old children, while no such association was detected for total fat, saturated fatty acids (SFA), total n-3 PUFA, EPA, DHA, arachidonic acid, and cholesterol.^21^

The inflammatory status, induced by obesity, GDM or poor diet composition^22,23^ could unfavorably influence neurodevelopment.^24^ Therefore, by increasing the intakes of anti-inflammatory dietary nutrients like n-3 PUFA,^25,26^ it might be possible to lower the woman’s low-grade inflammatory state that could subsequently benefit her child’s neurodevelopment. Our aims were to investigate the extent to which (1) maternal obesity, including body fat percentage, and GDM, and (2) maternal diet during pregnancy using multiple methods, including an evaluation of dietary quality, fish consumption, individual nutrients, and dietary inflammatory potential impact on the neurodevelopment of 2-year-old children.

## Materials and methods

### Study design and participants

**Fig. 1:**
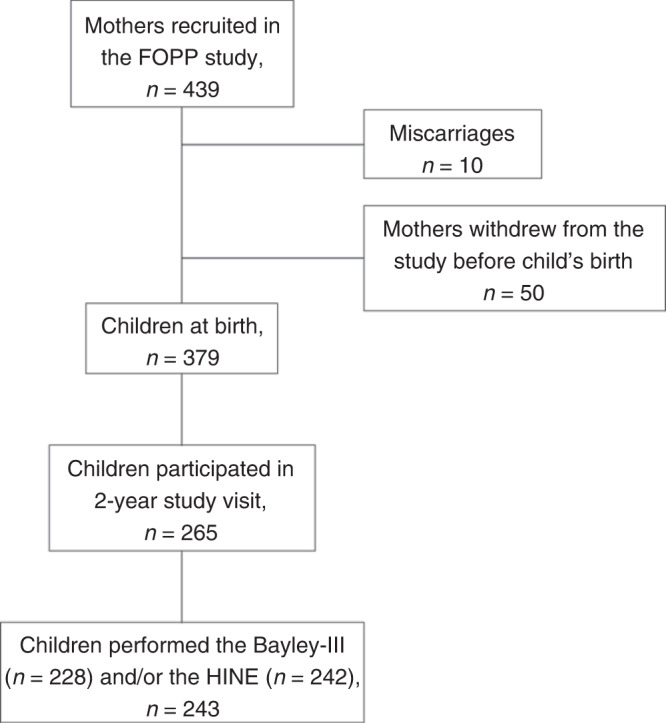
Flow chart of the present study. The data on neurodevelopmental assessments (the Bayley-III and/or the HINE) were available from 243 children.

We used data from a longitudinal, mother-child study described previously in detail^27^ to assess the neurodevelopment of 2-year-old children. These data were available from 243 children and their mothers (Fig. 1). The primary outcomes of the original double-blind, placebo-controlled randomized trial were to investigate if fish oil and/or probiotics supplements decrease the risk of GDM and allergies in children (ClinicalTrials.gov Identifier: NCT01922791). Here, our interest was the 2-year-old child’s neurodevelopment; since this was a predefined secondary outcome of the study, the impact of the intervention was not powered to detect statistically significant changes. However, this aspect has been explored (Supplementary Table S1) and considered in the analysis.

The inclusion criteria for the original study were pregnant women with overweight and who were in the early stages of their pregnancy (<18 gestational weeks). The exclusion criteria were GDM in the current pregnancy diagnosed before entry to the study; multifoetal pregnancy; and the presence of metabolic or inflammatory diseases, however, the presence of allergy was allowed. A total of 439 pregnant women were recruited in early pregnancy between October 2013 and July 2017 in Southwest Finland. The mothers were followed through their pregnancies and then both mothers and children for 2 years after delivery. The study was carried out according to the guidelines of the Declaration of Helsinki, and each woman provided written informed consent before participation. The study protocol was accepted by the Ethics Committee of the Hospital District of South-West Finland.

At baseline, the women were randomized into four intervention groups (fish oil + placebo, probiotics + placebo, fish oil + probiotics, placebo + placebo). A detailed description of the intervention has been published earlier.^27^ The intakes of EPA (0.22 g) and DHA (1.9 g) from fish oil capsules were added to the intakes from the diet in late pregnancy (total intake). Because probiotics do not include nutrients, they were not considered in the calculation of dietary intakes.

During the early (mean 13.9, SD 2.08 gestational weeks) and late pregnancy study visits (mean 35.1, SD 0.96 gestational weeks) the women’s dietary intakes were assessed and their body composition was measured. The background characteristics of the mothers (e.g., age, education, smoking) were collected by interviews and questionnaires during the early pregnancy study visit. Information regarding delivery and neonatal measurements was obtained from hospital medical records. The duration of breast-feeding was inquired from hospital medical records and interviews during the study visits (three, six, 12, and 24 months postpartum).

### Maternal diet

Dietary intake was recorded by three-day food diaries. The mothers were instructed both orally and in writing on how to record their consumption of food and drinks. A portion picture booklet was used to estimate the correctness of portion sizes. Mean daily intakes of energy and energy-yielding nutrients (Supplementary Fig. S1) were calculated with a computerized software (AivoDiet 2.0.2.3; Aivo, Turku, Finland). Maternal fish consumption was assessed by a questionnaire. The mothers were asked how many times they had eaten fish over 2 weeks prior to each study visit.

The overall quality of diet was assessed two times during pregnancy with the validated Index of Diet Quality (IDQ) questionnaire.^28^ The IDQ includes 18 questions regarding the frequency and amount of consumption of food products during the preceding week. The following criteria defined the healthy diet: use of wholegrain bread (≥4slices/day), saturated/unsaturated fatty acids (vegetable-oil-based margarine on bread, fish ≥2 times/week, low-fat ≤1% dairy products, vegetable-oil-based salad dressing), dairy products (≥4 dl/day), vegetables, fruits and berries (≥400 g/day), and sugar-containing drinks and sweets (soft drinks and sweets ≤1/week, fruit/berry juices ≤1glass/day), and <2 skipped meals/week. After scoring the questions, the dietary quality was defined to be good if the scores were ≥10 and poor if the scores were <10 out of the maximum 15 scores as depicted in the article describing the validation of the index.^28^

The Dietary Inflammatory Index (DII^®^), designed to assess diet-associated inflammation, is based upon findings in 1943 peer-reviewed articles that identified a total of 45 food parameters in relation to six specific inflammatory markers (IL-1β, IL-4, IL-6, IL-10, TNF-α, and CRP).^29,30^ Briefly, the scoring algorithm uses a global reference database (food consumption from eleven populations globally), and food parameter-specific inflammatory effect scores to create an individual’s overall DII score. DII scores range from strongly anti-inflammatory (−8.87) to strongly pro-inflammatory (+7.98). DII scores were derived from the three-day food diaries. A Z-score for each food parameter for each participant was calculated by subtracting the global mean from the self-report and then dividing this value by the standard deviation. The Z-scores were converted to a proportion to minimize the effects of outliers (“right-skewing”). The proportion (values 0–1) was centered by doubling and subtracting 1. These were then multiplied by the inflammatory effect score of each food parameter and summed to obtain an overall DII score for every participant in the study. A total of 28 food parameters were available from the food diaries in this study for computing the overall DII scores: energy, carbohydrate, protein, total fat, alcohol, fiber, cholesterol, SFA, MUFA, PUFA, n-3 and n-6 fatty acids, trans-fatty acids, niacin, thiamine, riboflavine, vitamins B12, B6, A, C, D, and E, iron, magnesium, zinc, selenium, folic acid, beta-carotene. We also computed the energy-adjusted DII (E-DII^TM^). This applies the same procedure as for the DII except it is utilized in the energy-adjusted global comparative database. Only 27 food parameters were used in the calculation of the E-DII scores because energy is in the denominator.

### Maternal obesity and GDM

Pre-pregnancy BMI (kg/m^2^) was calculated based on the woman’s self-reported pre-pregnancy weight and height measured during the first study visit with a wall stadiometer to the nearest 0.1 cm. The accuracy of self-reported weight was confirmed by a high correlation coefficient between self-reported weight and weight measured at the first study visit (*r* = 0.97, *p* < 0.001). Mothers were classified as having overweight or obesity based on their pre-pregnancy BMI. The mother’s adiposity was investigated in more detail by measuring their body composition using air displacement plethysmography (the Bod Pod system, software version 5.4.0, COSMED, Inc., Concord, CA) according to the manufacturer’s instructions. Thoracic gas volume was measured when possible and if not available, predicted thoracic gas volume was used in body composition calculations (first visit *n* = 21 (9%) and second visit *n* = 16 (7%)). After overnight fasting, cessation of drinking ≥4 h, and emptying the bladder, subjects entered the measurement chamber wearing a tight cap and tight underwear. They were advised not to exercise or to shower on the morning of the measurements. Proportion of fat (%) was calculated from density using the formulas devised by van Raaij et al.,^31^ which take into account the length of gestation and the presence of marked general swelling when necessary.

GDM was diagnosed with a 75 g 2-h oral glucose tolerance test according to Finnish current care guidelines^32^ that are in line with the American Diabetes Association 2007 guidelines. The test was offered in the health care clinics and was conducted in mid-pregnancy (median 25.9 gestational weeks, IQR 25.1–26.9) or in early pregnancy (median 14.9 gestational weeks, IQR 13.3–16.9) for women at risk for GDM.^32^ A total of 237 mothers provided an available test result from the oral glucose tolerance test at any stage of pregnancy.

### Assessment of neurodevelopment at 2 years of age

Children were assessed with the cognitive, language and motor scales of the Bayley Scales of Infant and Toddler Development–Third Edition (Bayley-III).^33^ The language scale includes subscales for receptive and expressive communication and the motor scale includes fine motor and gross motor subscales. The assessments were performed by trained psychology students or a physiotherapist (gross motor subscale). According to the manual, index scores (mean = 100, SD = 15) were calculated for the cognitive, language and motor composite scales, and standard scores (mean = 10, SD = 3) for the language and motor subscales. For children born prematurely (*n* = 13), <37 gestational weeks, corrected age was used instead of chronological age.

The structured neurological examination was performed by a trained physiotherapist using the Hammersmith Infant Neurological Examination (HINE).^34^ This consists of three sections: (1) Neurologic examination, (2) Developmental milestones, and (3) Behavior. The first section includes 26 items evaluating five subsections: cranial nerve function, posture, movements, tone and reflexes. Each item of the first section is scored individually, and these item scores are summed up to calculate the subsection scores and then the global score (minimum = 0, maximum = 78). The second section includes eight items describing developmental milestones, and the third section includes three items evaluating behavior during the assessment, but these are not included in the global score. The global score of HINE was categorized into optimal (≥74) or suboptimal scores (<74) according to the publication describing the optimality scores of HINE, after excluding the children born prematurely (*n* = 13).^34^

### Statistical analysis

The data were checked visually with histograms, and skewness <1 was used to determine normality. Normally distributed variables are summarized as means and standard deviations whereas those that were not normally distributed are described as medians and interquartile ranges. Normally distributed data were compared with Independent Samples t-test otherwise Mann–Whitney *U* test was used. One-Way ANOVA or Kruskall–Wallis H was used when comparing more than two groups. Categorical variables were cross-tabulated and a Chi-squared test or Fisher’s exact test was used to analyze data.

We applied general linear models and logistic regression models to investigate if maternal GDM, obesity, and IDQ could affect the child’s neurodevelopment (Bayley scores or suboptimal/optimal score of HINE) after adjustments for confounders. Variables were natural log-transformed for the linear model analysis if they were not normally distributed. Associations between body fat percentage, dietary variables, and the neurodevelopmental variables were assessed with Pearson Partial correlation coefficient or Spearman Partial correlation coefficient. The confounders were selected based on the group differences or previous knowledge on the factors affecting neurodevelopment. Furthermore, the effect of the intervention with fish oil and/or probiotics was considered in the analysis (Supplementary Table S1). Analysis were adjusted for maternal education, employee status, marital status, pre-pregnancy smoking status, primiparity, child’s sex, and pre-pregnancy BMI (except for analysis with obesity and body fat percentage). Models with GDM diagnosis as an independent variable were further adjusted for gestational weeks at delivery and models with obesity status as an independent variable for the child’s age at assessment and GDM status as these differed between the groups. The correlations between neurodevelopment variables and nutrient intakes were adjusted for multiple comparisons using the adaptive Benjamini–Hochberg procedure for the false discovery rate, *p* < 0.05 considered significant. All the analyses were performed with IBM SPSS statistics version 26.0 for Windows (IBM SPSS Inc. Chicago, IL) with a two-tailed *p* < 0.05 considered statistically significant.

## Results

### Clinical characteristics

The clinical characteristics of the mothers and their children (*n* = 243) are shown in Table 1. The majority of the women had a college or university education, and GDM was diagnosed in nearly every third woman. Approximately 61.3% of the women were classified as having overweight and the other 38.7% with obesity. The mean maternal body fat percentage in early pregnancy was 42.9 ± 5.5 and in late pregnancy 40.4 ± 5.1. Most children were born full term (94.7%). The majority of the children were born with a normal birth weight, but 2.5% were classified as small for gestational age (≤−2SD) and 5.8% were classified as large for gestational age (≥2 SD or >4500 g).

**Table 1:** Clinical characteristics of all children who participated in the Bayley-III and/or HINE assessments at the age of 2 years and their mothers, and according to GDM diagnosis (non-GDM/GDM) and pre-pregnancy overweight or obesity status.

Characteristics	All		Non-GDM	GDM	*p*^d^		Overweight	Obese	*p*^d^
	*n* = 243	*n*				*n*			
Maternal age 1st visit (years)^a^	30.9 ± 4.6	169/68	30.7 ± 4.5	31.5 ± 4.8	0.25	149/94	30.8 ± 4.1	31.0 ± 5.3	0.68
Maternal college or university education^b^	169 (70)	169/68	126 (75)	41 (60)	0.04	149/94	104 (70)	65 (69)	1.00
Married or common-law marriage^b^	235 (96.7)	169/68	165 (97.6)	64 (94.1)	0.23	149/94	145 (97.3)	90 (95.7)	0.71
Employee^b^	194 (79.8)	169/68	136 (80.5)	53 (77.9)	0.72	149/94	119 (79.9)	75 (79.8)	1.00
Pre-pregnancy BMI	29.4 ± 3.8^a^	169/68	28.3 (26.2; 30.8)^c^	30.1 (27.2; 33.3)^c^	0.002	–	–	–	–
Obese^b^	94 (38.7)	169/68	54 (32.0)	36 (52.9)	0.003	–	–	–	–
GDM^b^	68 (28.7)	–	–	–	–	147/90	32 (21.8)	36 (40.0)	0.003
Primiparity^b^	133 (54.7)	169/68	92 (54.4)	37 (54.4)	1.00	149/94	79 (53.0)	54 (57.4)	0.51
Smoking before pregnancy^b^	38 (15.6)	169/68	27 (16.0)	8 (11.8)	0.54	149/94	19 (12.8)	19 (20.2)	0.15
Smoking during pregnancy^b^	8 (3.3)	168/68	4 (2.4)	2 (2.9)	1.00	148/94	3 (2.0)	5 (5.3)	0.27
Gestational weeks at delivery^c^	40.0 (39.0–40.7)	169/68	40.1 (39.1; 40.9)	39.3 (38.3; 40.4)	0.003	149/94	40.0 (39.1; 40.9)	39.6 (38.7; 40.6)	0.15
Delivery <37 + 0 gestational weeks^b^	13 (5.3)	169/68	10 (5.9)	3 (4.4)	0.76	149/94	11 (7.4)	2 (2.1)	0.09
Small for gestational age^b^	6 (2.5)	169/68	4 (2.4)	2 (2.9)	1.00	149/94	3 (2.0)	3 (3.2)	0.68
Large for gestational age^b^	14 (5.8)	169/68	9 (5.3)	5 (7.4)	0.55	149/94	6 (4.0)	8 (8.5)	0.16
Unassisted vaginal delivery^b^	174 (71.6)	169/68	126 (74.6)	45 (66.2)	0.20	149/94	112 (75.2)	62 (66.0)	0.14
Child sex, girl^b^	119 (49.0)	169/68	82 (48.5)	35 (51.1)	0.77	149/94	74 (49.7)	45 (47.9)	0.79
Apgar 1 min^c^	9.0 (9.0–9.0)	169/68	9.0 (9.0; 9.0)	9.00 (9.0; 9.0)	0.81	149/94	9.0 (9.0; 9.0)	9.0 (9.0; 9.0)	0.25
Apgar 5 min^c^	9.0 (9.0–9.0)	169/67	9.0 (9.0; 10.0)	9.0 (9.0; 9.0)	0.87	148/94	9.0 (9.0; 9.0)	9.0 (9.0; 10.0)	0.54
Breast feeding period (months)^a^	11.6 ± 6.4	159/62	11.4 ± 6.1	12.4 ± 7.2	0.54	142/85	12.2 ± 6.2	10.5 ± 6.7	0.12
Child age at psychologist visit^a^	2.0 ± 0.03	163/62	2.0 ± 0.03	2.01 ± 0.03	0.07	144/87	2.0 ± 0.04	2.1 ± 0.03	0.02
Child age at physiotherapist visit^a^	2.0 ± 0.1	169/67	2.0 ± 0.04	2.0 ± 0.1	0.55	149/93	2.0 ± 0.1	2.0 ± 0.1	0.08

The differences in *n* are due to missing values. Overweight BMI 25–29.9 kg/m^2^, Obesity BMI ≥ 30 kg/m^2^.

*GDM* Gestational diabetes mellitus, *HINE* Hammersmith Infant Neurological Examination.

Data are expressed as ^a^mean ± SD, ^b^frequency (%) or ^c^median (interquartile range).

^d^Independent Samples *t*-test for normally distributed variables, Mann–Whitney *U* test for non-normally distributed variables and Fisher’s exact test for categorical variables.

Clinical characteristics of the women whose children participated and those who did not participate (including drop outs) in the Bayley-III and/or HINE assessments were similar in terms of age, pre-pregnancy BMI, and proportion of obese women, but the women whose children participated in the assessments had a higher education level, smoked less likely before pregnancy, and were more often primipara (Supplementary Table S2).

### Maternal GDM and obesity, and child’s neurodevelopment

On average, the children scored within the mean normative range in the Bayley-III. However, 6.2% (*n* = 27) of the children scored more than 1 SD below the mean on the expressive language subscale, 2.3% (*n* = 10) on the composite language scale, 1.3% (*n* = 3) on the cognitive scale, and 0.4% (*n* = 2) on the receptive language subscale.

A summary of the distributions of the children’s Bayley-III and HINE scores according to the maternal GDM and obesity status is presented in Table 2. Children born to mothers with GDM scored lower than children born to mothers without GDM on the expressive language scale, but did not differ in the other scales (adjusted models). No association was observed between maternal obesity and child’s neurodevelopment in the adjusted models (Table 2). When the impacts of GDM and maternal obesity on the child’s Bayley-III and HINE scores were examined together, they were associated with lower expressive language scores (adjusted model). The children of non-GDM + Obese mothers and GDM + Overweight mothers had a higher risk for a suboptimal performance in the HINE than the children of non-GDM + Overweight mothers (Supplementary Table S3).

**Table 2:** The Bayley-III scores and the global score of HINE of the 2-year-old children according to GDM diagnosis (non-GDM/GDM) and pre-pregnancy overweight or obesity status.

	*n*	Non-GDM adjusted mean (SE)	GDM adjusted mean (SE)	Adjusted mean difference (GDM vs non-GDM) / OR (95% CI)	Adjusted *p*^a^ or *p*	*n*	Overweight adjusted mean (SE)	Obese adjusted mean (SE)	Adjusted mean difference (obese vs overweigh) / OR (95% CI)	Adjusted *p*^a^ or *p*
Bayley-III										
Composite cognitive^b^	161/61	112 (2.69)	111 (2.85)	−1.39 (−5.36, 2.58)	0.49	143/85	113 (2.72)	109 (2.71)	−3.60 (−7.21; 0.01)	0.050
Composite language^b^	150/57	108 (3.13)	104 (3.33)	−4.34 (−9.07; 0.39)	0.07	136/76	107 (3.16)	105 (3.16)	−2.09 (−6.42; 2.24)	0.34
Expressive language^b^	150/59	10.2 (0.65)	9.09 (0.69)	−1.12 (−2.10; −0.15)	0.02	137/78	9.81 (0.66)	9.39 (0.65)	−0.42 (−1.31; 0.48)	0.36
Receptive language^b^	153/57	12.5 (0.57)	12.2 (0.60)	−0.30 (−1.15; 0.56)	0.50	137/78	12.5 (0.57)	12.1 (0.57)	−0.39 (−1.16; 0.39)	0.33
Composite motor^b^	126/45	112 (3.09)	109 (3.24)	−3.65 (−8.04; 0.74)	0.10	113/62	111 (3.09)	110 (3.10)	−0.87 (−4.83; 3.10)	0.67
Fine motor^b^	126/45	13.1 (0.63)	12.5 (0.66)	−0.64 (−1.54; 0.26)	0.16	113/62	12.9 (0.63)	12.5 (0.63)	−0.34 (−1.15; 0.48)	0.42
Gross motor^b^	169/66	11.8 (0.55)	11.2 (0.59)	−0.63 (−1.47; 0.21)	0.14	149/92	11.5 (0.56)	11.4 (0.56)	−0.07 (−0.82; 0.69)	0.86
HINE										
Global score^c,^*	169/66	76.0 (75.0; 77.0)	75.0 (74.0; 76.0)		0.08	149/92	76.0 (75.0; 77.0)	75.5 (74.0; 76.4)		0.10
Suboptimal/optimal score^d,e^	159/63	22 (13.8) 137 (86.2)	15 (23.8) 48 (76.2)	2.12 (0.92; 4.88)	0.08	138/90	20 (14.5) 118 (85.5)	18 (20.0) 72 (80.0)	1.32 (0.60; 2.91)	0.49

Optimal score of HINE ≥ 74, suboptimal score of HINE < 74. Overweight BMI 25–29.9 kg/m^2^, Obesity BMI ≥ 30 kg/m^2^.

*CI* Confidence interval, *GDM* Gestational diabetes mellitus, *HINE* Hammersmith Infant Neurological Examination, *OR* Odds ratio. The differences in *n* are due to missing values.

*Mann–Whitney *U* test for the Global score of HINE. Due to skewed distribution, adjusted analysis was conducted with categorical variable.

^a^General linear model. Adjusted for confounders. GDM as an independent variable: education, employee status, marital status, pre-pregnancy BMI, gestational weeks at delivery, pre-pregnancy smoking status, primiparity, child’s sex, and intervention groups. Obesity as an independent variable: education, employee status, marital status, GDM diagnosis, pre-pregnancy smoking status, primiparity, child’s sex and age at psychology assessment, and intervention groups.

Data are expressed as ^b^adjusted mean (SE), ^c^median (interquartile range) or ^d^frequency (%).

^e^Binary logistic regression model for categorical variable (Suboptimal score of HINE, non-GDM and overweight as reference categories).

To inspect maternal obesity in more detail, we measured their body fat percentage. Early pregnancy fat percentage correlated negatively with composite cognition (*r* = −0.16, adj.*p* = 0.02), expressive language (*r* = −0.14, adj.*p* = 0.046), composite motor (*r* = −0.16, adj.*p* = 0.03), and gross motor (*r* = −0.13, adj.*p* = 0.04) scores. Late pregnancy body fat percentage correlated negatively with composite cognition (*r* = −0.18, adj.*p* = 0.01) and receptive language (*r* = −0.15, adj.*p* = 0.03) scores.

### Maternal diet and child’s neurodevelopment

We assessed maternal diet composition using multiple methods. Maternal dietary fish consumption in early pregnancy (median 2.2, IQR 1.0–3.0 times/week) was not found to correlate with the child’s neurodevelopmental variables. A positive correlation was found between dietary fish consumption in late pregnancy (median 2.0, IQR 1.0–3.0 times/week) and expressive language scores (rho = 0.17, adj.*p* = 0.02).

When the mother’s overall dietary quality, assessed by IDQ, was utilized as a categorical variable (Table 3), a significant association was detected between an overall good dietary quality in late pregnancy and higher expressive language scores (adjusted model) (Table 3). No association was seen between early pregnancy IDQ and the child’s neurodevelopment. We did not detect any correlations between the children’s neurodevelopment and the maternal IDQ as a continuous variable (mean IDQ 9.6, SD 2.1) in early pregnancy or (mean 9.8, SD 2.0) in late pregnancy (adjusted models, data not shown).

**Table 3:** The Bayley-III scores and the global score of HINE of 2-year-old children by maternal diet quality measured by IDQ in early and late pregnancy.

		Early pregnancy					Late Pregnancy			
	*n*	Good diet quality adjusted mean (SE)	Poor diet quality adjusted mean (SE)	Adjusted mean difference (good-poor) / OR (95% CI)	Adjusted *p*^a^ or *p*	*n*	Good diet adjusted quality mean (SE)	Poor diet adjusted quality mean (SE)	Adjusted mean difference (good-poor) / OR (95% CI)	Adjusted *p*^a^ or *p*
Bayley-III										
Composite cognition^b^	107/119	110 (2.71)	112 (2.64)	−1.43 (−4.87; 2.00)	0.41	124/99	112 (2.65)	110 (2.65)	2.49 (−0.92; 5.90)	0.15
Composite language^b^	104/109	107 (3.21)	106 (3.11)	1.18 (−2.98; 5.34)	0.58	117/90	108 (3.13)	104 (3.15)	3.92 (−0.25; 8.10)	0.07
Expressive language^b^	102/11	10.1 (0.67)	9.48 (0.65)	0.60 (−0.27; 1.46)	0.18	119/91	10.1 (0.65)	9.25 (0.66)	0.87 (0.004; 1.73)	0.049
Receptive language^b^	102/109	12.3 (0.58)	12.5 (0.56)	−0.17 (−0.92; 0.57)	0.65	120/90	12.5 (0.57)	12.2 (0.57)	0.33 (−0.42; 1.08)	0.39
Composite motor^b^	83/90	110 (3.30)	112 (3.05)	−2.08 (−5.88; 1.72)	0.28	92/79	111 (3.24)	111 (3.13)	0.23 (−3.65; 4.11)	0.91
Fine motor^b^	120/90	12.8 (0.67)	13.0 (0.62)	−0.22 (−0.99; 0.55)	0.57	92/79	13.0 (0.66)	12.7 (0.64)	0.32 (−0.48; 1.12)	0.43
Gross motor^b^	87/123	11.4 (0.58)	11.8 (055)	−0.39 (−1.13; 0.36)	0.31	133/101	11.6 (0.57)	11.7 (0.56)	−0.05 (−0.81; 0.71)	0.90
HINE										
Global score^c,^*	115/124	76.0 (75.0; 77.0)	75.5 (74.0; 76.5)		0.27	133/102	76.0 (74.8; 76.5)	76.0 (74.0; 77.0)		0.86
Suboptimal/optimal score^d,e^	105/121	18 (17.1) 87 (82.9)	19 (15.7) 102 (84.3)	0.84 (0.39; 1.81)	0.66	125/100	20 (16.0) 105 (84.0)	16 (16.0) 84 (84.0)	0.93 (0.42; 2.05)	0.86

Early pregnancy, mean 13.9, SD 2.08 gestational weeks; late pregnancy mean 35.1, SD 0.96 gestational weeks. Optimal score of HINE ≥ 74, suboptimal score of HINE < 74.

*CI* Confidence interval, *HINE* Hammersmith Infant Neurological Examination, *OR* Odds ratio.

The differences in *n* are due to missing values.

*Mann–Whitney *U* test for the Global score of HINE. Due to skewed distribution, adjusted analysis was conducted with categorical variable.

^a^General linear model. Adjusted for maternal education, employee status, marital status, pre-pregnancy BMI, pre-pregnancy smoking status, primiparity, child’s sex, and intervention groups.

Data are expressed as ^b^adjusted mean (SE), ^c^median (interquartile range) or ^d^frequency (%).

^e^Binary logistic regression model for categorical variable (suboptimal score of HINE, poor diet quality as a reference category).

The inflammatory potential of the maternal diet was evaluated by the DII and E-DII scores. The mean DII score in early pregnancy was 0.66 ± 1.77 (E-DII mean −1.23, SD 1.63) and in late pregnancy −0.71 ± 1.70 (E-DII mean −1.18, SD 1.68). No association was seen between inflammatory potential and child’s neurodevelopment (adjusted models, data not shown). To investigate if the overall dietary quality was associated with the diet’s inflammatory properties, we calculated correlations between the IDQ and DII/E-DII. It was found that the IDQ correlated negatively with the DII and E-DII in early (*r* = −0.40, *p* < 0.001, *r* = −0.40, *p* < 0.001) and in late pregnancy (*r* = −0.25, *p* < 0.001, *r* = −0.25, *p* < 0.001), respectively.

We also investigated the mother’s dietary intake in more detail by evaluating nutrient intakes, and detected modest correlations with the child’s neurodevelopment after adjusting for confounders (Supplementary Fig. S1). A positive correlation was seen between the intake of carbohydrate in late pregnancy and gross motor skills. Furthermore, negative correlations were detected between maternal intakes of total fat, MUFA, PUFA, DHA, and n-6 FA in late pregnancy and fine motor skills. No significant correlations were seen between nutrient intakes in early pregnancy and neurodevelopmental variables. After adjusting for multiple comparisons, the correlations between intakes of total fat, PUFA, MUFA, and n-6 FA in late pregnancy and fine motor scores remained statistically significant (Supplementary Fig. S1).

## Discussion

In this longitudinal study, we demonstrated that 2-year-old children of mothers without GDM and/or obesity displayed a more favorable neurodevelopment compared to those of mothers with GDM and/or obesity, although the scores in developmental assessments were within the mean normative range in both groups. Furthermore, we found that maternal higher fish consumption and good dietary quality were associated with more favorable neurodevelopment of children.

Our study, together with previous findings indicate that GDM may exert an unfavorable impact on the child’s neurodevelopment. We observed that 2-year-old children of mothers without GDM had better expressive language skills compared to the children of mothers with GDM. Our finding is somewhat in line with a report, although using different methods for the neurodevelopment evaluation, indicating that 1.5- and 7-year-old children of mothers with GDM had poorer expressive language skills when compared to those of mothers without GDM.^10^ The mechanism to explain how GDM affects child’s neurodevelopment is not completely understood, but may relate to the epigenetic modifications caused by hyperglycemia.^35^ Another plausible mechanism is that DHA transfer from mother to foetus is lowered in women with GDM.^36^ It is noteworthy that also other factors, including maternal socio-economic status, which were accounted for in our analyses, contribute to a child’s neurodevelopment.

We believe that this is the first study investigating the association between maternal body fat percentage and her child’s neurodevelopment. Our novel finding was that a lower maternal body fat percentage associated with better cognition, language and motor skills, whilst only a tendency was seen for the better skills in the cognitive scale in children of mothers with overweight than those of mothers with obesity as based on pre-pregnancy BMI. Our results are supported by previous investigators indicating that children of mothers with obesity have poorer mental development, cognition, communication, problem-solving, and motor skills at 11–42 months compared to those of mothers with normal weight.^37–39^ When evaluating the effects of GDM and obesity together, we found that children of mothers with both obesity and GDM had weaker expressive language skills as compared to those of mothers without GDM and obesity. Although the scores were in the normal range, our findings are interesting and suggest that GDM, particularly in mothers with obesity, could lead to poorer child’s neurodevelopment calling for larger studies to verify this proposal. It is possible that a systemic low-grade inflammation could partly explain the association between maternal obesity, GDM, and child’s poorer neurodevelopment. Pregnancy itself increases the level of systemic low-grade inflammation in the body, and in women with obesity the levels of inflammatory markers, such as cytokines are elevated even more.^40^ Thus, the children of these mothers are likely to be exposed to inflammation during pregnancy as the placentas of mothers with obesity have been shown to contain elevated levels of pro-inflammatory markers.^40^ This concept also is supported by prior studies indicating that a higher maternal serum concentration of the pro-inflammatory cytokine IL-1 was associated with externalizing and internalizing symptoms and lower general conceptual abilities in their children.^41,42^

We found that the children of mothers with an overall good dietary quality had superior language skills than those of mothers with an overall poor dietary quality. When we evaluated maternal diet during pregnancy in more detail, it was found that higher fish consumption from diet correlated with better expressive language skills in the children. Our findings are in line with previous reports^20,21,43,44^ emphasizing that an overall good dietary quality seems to be beneficial for the child’s neurodevelopment. The potential beneficial effects of a good quality diet may relate to its nutritional content. Vegetables, fruits, and berries include vitamins, such as folate, while fish is rich of n-3 PUFA and iodine. These nutrients are known to be important for child’s neurodevelopment.^45–47^ Furthermore, a good quality diet may excert anti-inflammatory effects e.g., due to n-3 PUFA.^26^ In contrast, a poorer diet composition, including higher intake of fat, especially SFA, increases the low-grade inflammation in the body.^22^ This concept is partly supported by our finding that IDQ and DII correlated negatively with each other, indicating that the diet of mothers with an overall good quality of diet is less inflammatory. However, when using the adjusted models, we did not detect an association between maternal dietary inflammatory potential and the child’s neurodevelopment. The reason for this finding is not clear, but may relate to the number of subjects studied as in a larger study, with 68479 subjects, a pre-conceptional anti-inflammatory diet decreased the risk of impaired neurodevelopment in 3-year-old children.^48^

We further examined the maternal intake of individual nutrients, and found that a higher intake of total fat correlated negatively with fine motor skills in children. In more detail, negative correlations were seen for MUFA, PUFA, DHA, and n-6 PUFA. As n-6 PUFAs generally have pro-inflammatory effects in the body,^49^ this finding is in line with our assumption that inflammation could partly explain the association between nutrient intakes and poorer child’s neurodevelopment. The negative correlation between DHA intake and fine motor skills was surprising as DHA is needed in brain development.^47^ Previous studies have shown that n-3 PUFA intake or supplementation may improve a child’s neurodevelopment, such as problem-solving and motor skills.^19,50^ The explanation for our finding remains unknown, but may relate to the fact that all of the women had overweight or obesity. As obesity is associated with higher level of systemic low-grade inflammation, it is possible that the putative benefits of n-3 PUFA were diminished. This has been shown in a recent study, where n-3 PUFA administration led to a lower plasma concentration of n-3 PUFA in women with obesity as compared to women with normal weight.^51^

The strengths of our study are in the prospective study design and the detailed data collection within a clinical study setting that allowed us to take into account multiple confounding factors in the statistical analysis, these including maternal socioeconomic status and smoking habits. Furthermore, we used a comprehensive assessment of maternal diet: food diaries, diet quality index, fish consumption questionnaire, and diet inflammatory potential, which is a validated tool also with a lower number of parameters,^29,52^ combined with valid and structured neurodevelopment assessment methods (Bayley-III and HINE) that have also been utilized in previous studies.^12,53^ Women’s obesity and overweight were determined by pre-pregnancy BMI and their body composition was measured by a robust method, air displacement plethysmography, equivalent to the gold standard method of body composition measurements, that provides a more detailed way to evaluate adiposity, although not separating fat distribution, i.e., visceral and subcutaneous fat or foetal and maternal tissues.^54^ To our knowledge, no previous studies have investigated the association between maternal body fat percentage and the child’s neurodevelopment.

We did not enroll women with normal weight, which is one limitation of this study; this is due to the fact that we chose to investigate a risk group of pregnant women for metabolic complications. It is noteworthy that overweight is common in pregnant women in Finland^3^ and globally,^1^ thus our study population actually represents a very common group of clients in maternal welfare clinics. A second limitation is that the neurodevelopment was a predefined secondary outcome of the study and therefore no power calculations were conducted for the present study; thus, unfortunately, our intervention was not powered to detect differences in child’s neurodevelopment. But both fish oil and probiotics,^50,55^ and importantly their combination might be beneficial for neurodevelopment and this hypothesis deserves further investigation. One further limitation is the lack of Finnish normative values for the index and standard scores in Bayley-III complicating the interpretation of our results. During the development of the Finnish version of the Bayley-III, a Finnish sample was assessed and the performance of the Finnish children at 2 years of age was shown to be overall somewhat superior to the test norms, particularly the receptive language scores. The test has nevertheless been used in previous studies with Finnish study populations.^53,56^ We used a previously validated IDQ^28^ a short method to describe the overall quality of diet, which might not compass all aspects of the dietary quality. A further limitation is that the mothers whose children participated in the neurodevelopment assessments, had a higher education level compared to the mothers whose children did not participate, although education was taken into account as a confounder in the analysis. It is noteworthy that along with pregnancy circumstances, the environment after delivery, including the mother’s and child’s lifestyle and diet, such as child feeding, contribute to neurodevelopment. A further limitation is that we were not able to adjust the analysis for breast-feeding since this data were not available from all the mothers. In fact, not all of the previous studies have considered breast-feeding when evaluating child’s neurodevelopment,^10,12,19,43^ although some other reports have.^39,44^ Lastly, although we assessed diet-induced inflammation, we did not investigate the inflammatory status of the mothers.

## Conclusions

In conclusion, child’s neurodevelopment is influenced by various maternal factors already during pregnancy. GDM and higher maternal adiposity, measured here by air displacement plethysmography, may have unfavorable effects on 2-year-old children’s language, cognitive, and motor skills, although the neurodevelopmental performance of these children was in the normal range, and thus the clinical significance of the finding remains to be further elucidated. A good dietary quality and fish consumption during pregnancy were associated with more favorable language skills of children. Our findings emphasize that maternal metabolic health and even subtle changes in dietary quality and composition during pregnancy may influence the child’s neurodevelopment.

## Supplementary Information

Supplementary material

## Data Availability

The datasets analysed during the current study are not publicly available due to their containing information that could compromise participant privacy and consent.
