# Supplementary material for: Maternal obesity, gestational diabetes mellitus, and diet in association with neurodevelopment of 2-year-old children
Source: Pediatr Res. 2023 Jan 3;94(1):280–9. doi: 10.1038/s41390-022-02455-4 (PMC10356612; doi:10.1038/s41390-022-02455-4)
Supplement: Supplementary file 1 — Supplementary material [file 41390_2022_2455_MOESM1_ESM.pdf]

Supplementary material

Maternal obesity, gestational diabetes mellitus, and diet in association with neurodevelopment of 2-year-old children

Lotta Saros, loevpa@utu.fi, corresponding author

Institute of Biomedicine, Research Centre for Integrative Physiology and Pharmacology, University of Turku, 20520 Turku, Finland

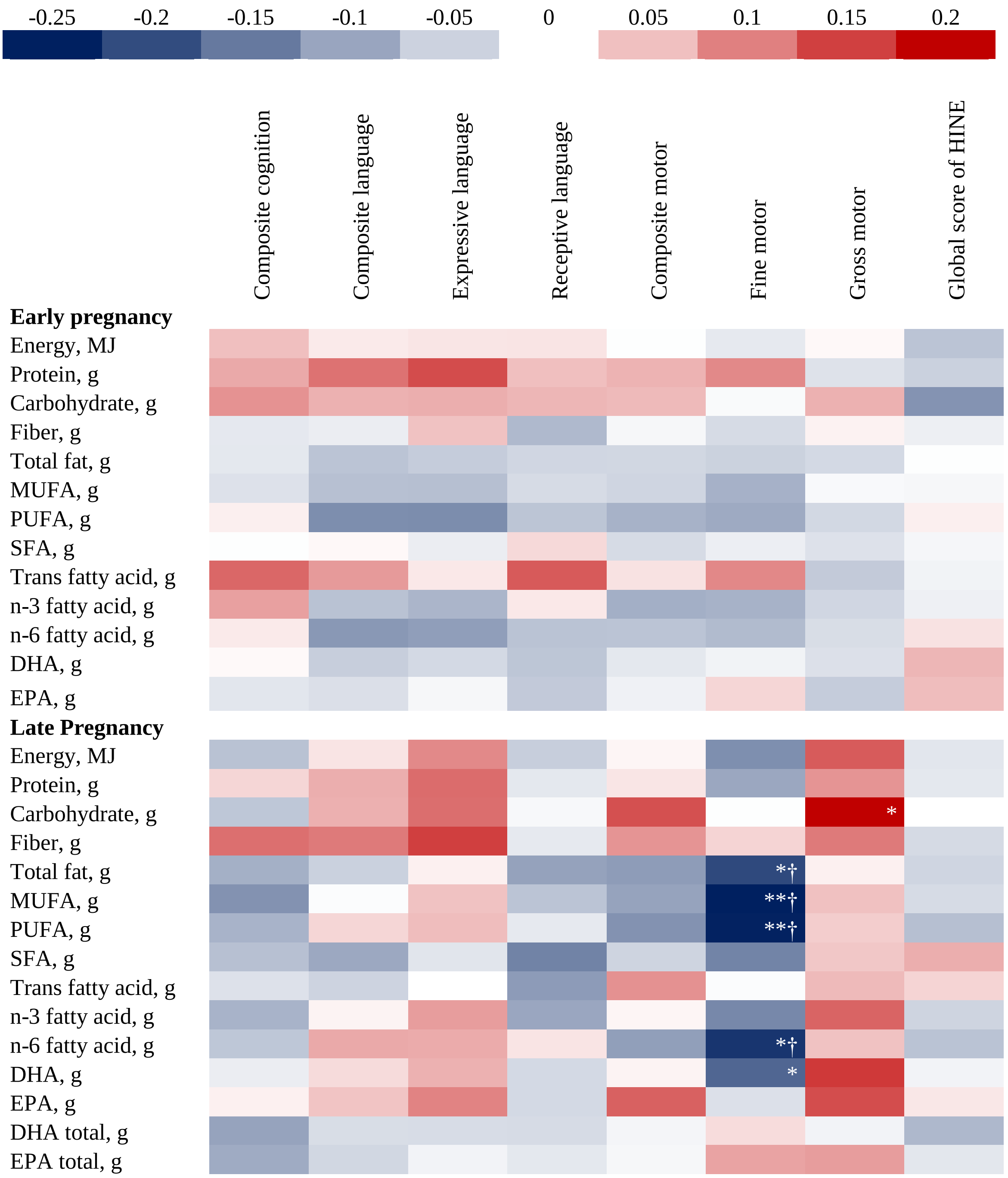

Supplemental Figure S1. Heatmap showing correlations between maternal energy and nutrient intakes in early and late pregnancy and child's Bayley-III scores and the global score of HINE at the age of two years. The intensity of the colors represents the degree of Pearson's or Spearman's correlation coefficient, red color indicating a positive correlation and blue indicating a negative correlation (*p<0.05, **p<0.01). Analysis adjusted for maternal education, employee status, marital status, pre-pregnancy BMI, primiparity, pre-pregnancy smoking status, and child's sex. After multiple correction

with Benjamini-Hochberg procedure, the correlations between intakes of total fat, MUFA, PUFA, and n-6 fatty acid in late pregnancy and fine motor scores remained significant (corrected †p<0.05).

DHA, Docosahexaenoic acid; EPA, Eicosapentaenoic acid; HINE, Hammersmith Infant Neurological Examination; MUFA, Monounsaturated fatty acid; PUFA, Polyunsaturated fatty acid; SFA, Saturated fatty acid; EPA total and DHA total, intake from diet and intervention supplements.

Supplemental Table S1 The Bayley-III scores and the global score of HINE of 2-year-old children by intervention groups.

	n	Fish oil	Probiotics	Fish oil and probiotics	Placebo	Adjusted p[†] or p
		Adjusted mean (SE) or geometric mean (95% CI)	Adjusted mean (SE) or geometric mean (95% CI)	Adjusted mean (SE) or geometric mean (95% CI)	Adjusted mean (SE) or geometric mean (95% CI)	
**Bayley-III**						
Composite cognitive[b]	59/53/57/59	108 (103;114)	111 (105; 117)	110 (105; 116)	113 (107; 118)	0.28
Composite language[a]	55/51/57/54	106 (3.56)	103 (3.50)	106 (3.44)	110 (3.29)	0.11
Expressive language[a]	56/51/53/55	9.69 (0.74)	9.00 (0.73)	9.54 (0.72)	10.6 (0.68)	0.045[**]
Receptive language[a]	56/52/52/55	12.3 (0.64)	12.1 (0.63)	12.5 (0.62)	12.8 (0.59)	0.54
Composite motor[b]	43/41/44/47	110 (104; 117)	109 (103; 115)	111 (105; 118)	113 (107; 119)	0.44
Fine motor[b]	43/41/44/47	12.9 (11.6; 14.5)	12.4 (11.1; 13.8)	12.6 (11.3; 14.1)	13.1 (11.9; 14.5)	0.50
Gross motor[a]	63/57/58/63	11.5 (0.59)	11.5 (0.62)	11.8 (0.62)	11.7 (0.59)	0.86
**HINE**						
**Global score[c]**	63/57/57/64	76.0 (74.0; 76.5)	76.0 (75.0; 76.5)	76.0 (74.0; 77.0)	76.0 (75.0; 76.9)	0.86
Suboptimal/<br>optimal score[d]	60/54/52/62	12 (20.0)<br>48 (80.0)	9 (16.7)<br>45 (83.3)	11 (21.2)<br>41 (78.8)	6 (9.7)<br>56 (90.3)	0.33
OR (95% CI)<br>Adjusted p [¥]		2.94 (0.97; 8.90)<br>0.06	1.72 (0.55; 5.38)<br>0.35	2.83 (0.93; 8.65)<br>0.07	1	

Data are expressed as [a] adjusted mean (SE) or [b] adjusted geometric mean (95% CI) and [c] median (interquartile range) or [d] frequency (%).

Composite cognitive, composite motor and fine motor scales ln transformed for the analysis due to skewed distributions.

Chi squared test for categorical variable. Optimal score of HINE ≥74, suboptimal score of HINE <74.

[†] General linear model or [¥] logistic regression model (suboptimal score of HINE, placebo group as a reference category) adjusted for maternal pre-pregnancy smoking status as it differed significantly between the intervention groups (data not shown) and education, employee status, marital status, primiparity, child's sex, and pre-pregnancy BMI.

[*] Mann Whitney U-test for Global score. Due to skewed distribution, adjusted analysis were conducted with categorical variable.

[**]Significant difference between probiotics and placebo (β= -1.64, 95% CI -3.21; -0.07, p=0.035) after Bonferroni correction.

The differences in n are due to missing values. HINE, Hammersmith Infant Neurological Examination.

Supplemental Table S2 Comparable baseline characteristics between the mothers whose children participated in the Bayley-III and/or HINE assessments and the mothers whose children did not participate (including drop outs).

**Characteristics**		**Participated in Bayley-III and/or HINE**	**Not participated in Bayley-III and/or HINE**	**p**[†]
	n*			
Maternal age 1st visit (years)[a]	243/195	30.9±4.6	30.2±4.5	0.14
Maternal college or university education[b]	243/148	169 (70)	70 (47)	<0.001
Married or common-law marriage[b]	243/149	235 (96.7)	138 (92.6)	0.09
Employee[b]	243/147	194 (79.8)	111 (75.5)	0.32
Pre-pregnancy BMI[c]	243/195	28.7 (26.4; 31.4)	28.7 (26.5; 33.0)	0.30
Obese[b]	243/195	94 (39)	78 (40)	0.84
Primiparity[b]	243/195	133 (55)	77 (40)	0.002
Smoking before pregnancy[b]	243/150	38 (16)	48 (32)	<0.001

Data are expressed as [a] mean ± SD, [b] frequency (%) or [c] median (IQR).

[†] Independent Samples t-test for normally distributed variables, Mann-Whitney U test for non-normally distributes variables, and Fisher's exact test for categorical variables.

*One excluded because of familial hypercholesterolemia
GDM, gestational diabetes mellitus; HINE, Hammersmith Infant Neurological Examination.
Overweight: BMI 25-29.9kg/m^2, Obese: BMI≥30 kg/m^2. The differences in n are due to missing values.

Supplemental Table S3 The Bayley-III scores and the global score of HINE of the 2-year-old children subdivided by GDM, (non-GDM/GDM) and pre-pregnancy BMI classified as overweight or obese.

	n	Non-GDM & overweight	Non-GDM & obese	GDM & overweight	GDM & obese	Adjusted p[†] or p
		Adjusted mean (SE) or geometric mean (95% CI)	Adjusted mean (SE) or geometric mean (95% CI)	Adjusted mean (SE) or geometric mean (95% CI)	Adjusted mean (SE) or geometric mean (95% CI)	
**Bayley-III**						
Composite cognitive[a]	112/29/49/32	113 (2.82)	111 (3.43)	110 (3.06)	108 (3.11)	0.16
Composite language[a]	106/28/44/29	109 (3.29)	105 (4.00)	107 (3.59)	102 (3.67)	0.18
Expressive language[a]	106/29/44/30	10.3 (0.68)	9.47 (0.82)	10.1 (0.74)	8.59 (0.75)	0.045[**]
Receptive language[a]	107/28/46/29	12.7 (0.59)	12.2 (0.72)	12.2 (0.65)	12.2 (0.66)	0.58
Composite motor[a]	88/23/38/22	112 (3.26)	110 (3.75)	112 (3.42)	106 (3.63)	0.21
Fine motor[a]	88/23/38/22	13.2 (0.67)	12.5 (0.77)	12.9 (0.70)	12.2 (0.74)	0.32
Gross motor[b]	115/32/54/34	11.4 (10.4; 12.5)	11.1 (9.95; 12.5)	11.6 (10.5; 12.8)	10.7 (9.63; 11.8)	0.34
**HINE**						
Global score[c, *]	115/32/54/34	76.0 (75.0; 77.0)	75.0 (73.5; 76.4)	75.5 (73.9; 76.5)	75.5 (74.0; 76.0)	0.08
Suboptimal/ optimal score[d]	107/29/52/34	10 (9.3) 97 (90.7)	9 (31.0) 20 (69.0)	12 (23.1) 40 (76.9)	6 (17.6) 28 (83.4)	0.02
OR (95% CI) Adjusted p [¥]		1	6.53 (2.09; 20.4) 0.001	3.27 (1.22; 8.78) 0.02	1.82 (0.52; 6.37) 0.35	

Data are expressed as [a] adjusted mean (SE) or [b] adjusted geometric mean (95% CI), and [c] median (interquartile range) or [d] frequency (%).

Gross motor scale ln transformed for the analysis due to skewed distribution.

Chi squared test for categorical variable. Optimal score of HINE ≥74, suboptimal score of HINE <74.

[†] General linear model or [¥] logistic regression model (suboptimal score of HINE, Non-GDM+overweight as a reference category) adjusted for maternal education, employee status, marital status, pre-pregnancy smoking status, primiparity, gestational weeks at delivery, child’s sex and age at psychology assessment, and intervention groups.

[*] Mann Whitney U test for Global score. Due to skewed distribution, adjusted analysis was conducted with categorical variable.

** Significant difference between non-GDM+overweigh and GDM+obese (β=1.74, 95% CI 0.04; 3.45, p=0.043) after Bonferroni correction.

GDM, Gestational diabetes mellitus; HINE, Hammersmith Infant Neurological Examination. Overweight BMI 25-29.9kg/m2, Obesity BMI≥30 kg/m2. The differences in n are due to missing values.
